# Non-occlusive mesenteric ischemia leading to '*pneumatosis intestinalis*': a series of unfortunate hemodynamic events

**DOI:** 10.1186/1757-1626-1-60

**Published:** 2008-07-25

**Authors:** Abhijeet Dhoble, Kamakshi Patel, Atul Khasnis

**Affiliations:** 1 Department of Internal Medicine, Michigan State University, East Lansing, Michigan, USA

## Abstract

**Background:**

Non-occlusive mesenteric ischemia (NOMI) is not uncommon in intensive care units. NOMI indicate ischemia of bowel wall without any significant obstruction in the mesenteric arteries. Common causes of NOMI include sepsis, severe cardiac failure, and any critical illness. Mesenteric circulation can suffer due to low cardiac output leading to very unfortunate outcomes. Pneumatosis Intestinalis is a radiologic sign which represent gas in the bowel wall, and could indicate mesenteric ischemia.

**Case Presentation:**

We present a fatal case of a patient who developed NOMI secondary to multiple factors. Patient died after a long protracted course in the hospital secondary to severe septic shock.

**Conclusion:**

This case emphasizes the importance of early detection and management of NOMI in a patient with low cardiac output and abdominal pain. In majority of the studies, NOMI is associated with high morbidity and mortality.

## Background

Non-occlusive mesenteric ischemia (NOMI) accounts for about 20% of all the cases of acute mesenteric ischemia (AMI), with mortality of about 70% [1-3]. Mesenteric circulation can suffer due to low cardiac output leading to very unfortunate events. '*Pneumatosis Intestinalis*' (PI) is a radiologic sign which represent gas in the bowel wall, and could indicate mesenteric ischemia [1]. Increased awareness about NOMI is very important amongst internists to improve the outcomes. NOMI is not uncommon in intensive care units (ICU), but is under recognized [2].

## Case presentation

A 55 years old African American man presented to the emergency room with progressive dyspnea, orthopnea, and peripheral edema. He also complained of severe epigastric pain which started few hours before presentation. His medical problems included dilated cardiomyopathy, type 2 diabetes mellitus, hypertension, hyperlipidemia, and polysubstance abuse. An implantable cardioverter defibrillator was implanted one year ago for the low ejection fraction (EF) of 25%. His medications included digoxin, lisinopril, amlodipine, aspirin, glipizide, atorvastatin, and furosemide. On examination, he had a S3, bi-basilar crackles, 3+ pitting edema in legs, mild abdominal wall rigidity, but no guarding or rebound tenderness. Otherwise examination was unremarkable. Laboratory studies were remarkable for marginal elevation of amylase and lipase (346 U/L and 155 U/L respectively), and brain natriuretic peptide of 1834 pg/ml. Blood urea nitrogen and creatinine were 79 mg/dl and 2.9 mg/dl respectively. Urine drug screen was positive for cocaine metabolites. He later on confirmed that he consumed cocaine six hours prior to his admission. Computed Tomogram (CT) scan of abdomen and pelvis did not show any acute abnormality on admission. Estimated EF on two-dimensional echocardiography was 15%.

**Figure 1:**
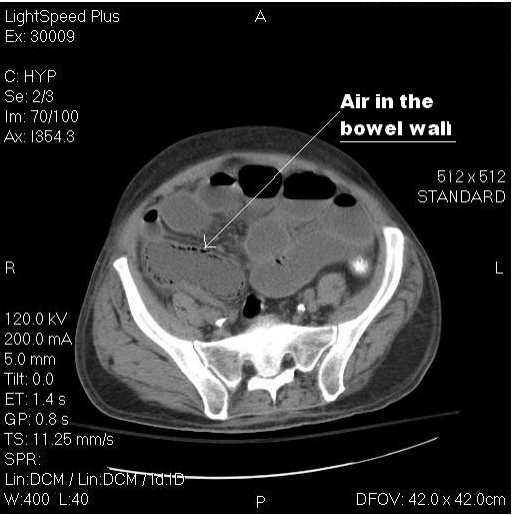
**Non contrast computed tomography of the abdomen and pelvis (axial view) shows air in the bowel wall.** This radiologic sign is called *Pneumatosis Intestinalis*. There is also evidence of an over-distended ileum, and collapsed left colon suggesting an intestinal obstruction.

He was started on treatment for exacerbation of congestive heart failure (CHF). Mesenteric ischemia was very high on differential due to multiple risk factors. Mild pancreatitis was also suspected, and a decision was made to monitor him clinically. He started feeling better on standard medical treatment. After two days, he developed severe abdominal pain and hypotension. A repeat non-contrast CT scan of the abdomen showed '*pneumatosis intestinalis' *(figure 1). He was promptly taken for the surgery. Proximal colon was found to be ischemic, and was resected. Mild atherosclerotic disease of the mesenteric arteries was noted. No thrombus or occlusion of the superior mesenteric artery was found at surgery. Patient was transferred to ICU. Pathological report of the resected bowel revealed features of NOMI. His further course in the ICU was complicated by progressive septic shock and death.

## Discussion

NOMI is usually caused by intense vasoconstriction in the setting of hypoperfusion of peripheral splanchnic arteries. Risk factors include low cardiac output state, sepsis, use of vasospastic drugs, digitalis, hemodialysis, major heart or abdominal surgery, and any critical illness [1,2]. CHF was found to be the major risk factor in one of the large population based study [3]. The common presentation is abdominal pain out of proportion to the physical signs. NOMI is frequently associated with pancreatitis due to proximity of superior mesenteric artery and celiac plexus to the pancreas [1].

This patient had very low EF at presentation, was on digoxin, and had recently consumed cocaine. All of these are risk factors for NOMI [1,2,4]. He had severe abdominal pain, and laboratory evidence of elevated pancreatic enzymes. But lack of any evidence of AMI on CT scan delayed the treatment of NOMI. Early recognition of NOMI is very important, and high clinical suspicion plays a major role in the prevention of unfortunate events. Delay in the identification is the major reason for high mortality in these patients. Intestinal ischemia is usually not well tolerated, and becomes decisive after 3–6 hours [1].

The purpose of this case report is to make internists aware about this entity, as NOMI is not uncommon, especially in critically ill patients. Internists are usually the gatekeeper for such patients, and early recognition of symptoms and signs can prevent series of unfortunate events. Prompt referral to gastroenterology and surgery is imperative. Despite of non invasive methods such as CT and ultrasound, angiography is still gold standard [1].

This patient had multiple risk factors to develop NOMI, and we speculate that all of them acted synergistically. These types of patients need judicious use of vasoactive drugs, diuretics, and fluids, especially considering history of CHF. Therefore invasive hemodynamic monitoring and early involvement of specialist care can make a difference in the outcome.

## Conclusion

Early recognition of NOMI is very important, and high clinical suspicion plays a major role in the prevention of unfortunate events.

## Competing interests

The authors declare that they have no competing interests.

## Authors' contributions

All authors contributed equally in collecting patient data, chart review, and editing medical images. All authors read and approved the final manuscript.

## Consent

An informed consent was obtained from the patient's sister for publication of this case report and accompanying images in *cases journal*. A copy of the written consent is available for review by the Editor-in-Chief of this journal.
